# Advances in Chronic Kidney Disease

**DOI:** 10.3390/ijms17081314

**Published:** 2016-08-11

**Authors:** Alan R. Parrish

**Affiliations:** Department of Medical Pharmacology and Physiology, School of Medicine, University of Missouri, Columbia, MO 65212, USA; parrishar@health.missouri.edu; Tel.: +1-573-884-4391

Chronic kidney disease (CKD) is characterized by renal dysfunction that is present for more than 3 months; it is also associated with a number of comorbidities [1,2]. There are a number of causes of CKD, most notably type 2 diabetes and hypertension [3,4,5,6,7]. CKD is staged using estimated glomerular filtration rate (eGFR) and albuminuria [1,2]. These stages range from an asymptomatic condition (eGFR > 60 mL/min/1.73 m^2^ and urinary albumin < 30 mg/g) to end-stage renal disease (ESRD) (eGFR < 15 mL/min/1.73 m^2^ and urinary albumin > 300 mg/g), which requires dialysis or transplantation. The prevalence of CKD is significant, ranging from 2.5% to 11.2% of adults in Europe, Asia, North America and Australia [8]. In the United States the prevalence was reported to be 13.7% between 2007 and 2012 [9] and it is predicted that the prevalence will increase to 14.4% in 2020 and 16.7% in 2030 [10]. The progression of CKD is variable, necessitating further research into factors that accelerate or attenuate the disease, as well as the pathogenic mechanisms that underlie the heterogeneity of the CKD progression. CKD patients also experience poorer quality of life and loss of function compared to healthy individuals [11,12,13]. While directly assessing the economic burden of CKD and ESRD is difficult, the medical cost of patients with stage 4 and 5 CKD not requiring dialysis ranges from $7,000 to $65,000 annually, with the yearly medical care cost of ESRD patients estimated at $65,000 (Medicare) to $96,000–$180,000 (private insurance) per patient [14]. Worldwide, it is estimated that 1 trillion dollars is spent annually on ESRD patients [7], indicating the tremendous impact of this disease on health care costs.

This Special Issue contains papers addressing a number of important issues in CKD with a major focus on: (1) mechanisms of pathogenesis; and (2) therapeutic interventions. In terms of mechanisms, a review focuses on the role of receptor tyrosine kinases (RTKs), most notably growth factor RTKs, in the progression of CKD [15]. In this article the potential of RTK inhibitors as therapeutic agents is also addressed. The role of autophagy and the innate immune response in the pathology of acute kidney injury (AKI) is also reviewed [16]. Given that AKI often occurs with a background of CKD [17], and that AKI can lead to CKD [18], this review may stimulate research into the role of these mechanisms in the relationship between acute and chronic renal dysfunction. The AKI–CKD connection is further investigated in a study showing that toll-like receptor 4 (TLR4) knockout mice are protected against AKI, but not fibrosis post ischemic injury [19]. Microvascular rarefaction after injury, however, was attenuated. Angiotensin II induces dipeptidyl protease 4 (DPP4) concurrent with suppression of megalin expression; this may have an important role in progression of obesity related renal dysfunction. The molecular pathway linking these pathways is elucidated in an elegant study [20]. The expression of kidney injury molecule-1 in proximal cysts in a rat polycystic kidney disease (PKD) model (PKD/Mhm) suggests that it may play a role in disease progression [21]. An interesting study demonstrates an inverse relationship between endometriosis and CKD, an effect that is abrogated by menopause [22]. These results should stimulate further research on the role of hormones in CKD. Proteomic analysis revealed that proteins involved in inflammation, coagulation, vascular damage and calcification are altered in atherosclerosis-related CKD and provide important data to examine the pathogenesis, as well as therapeutic targets, for this CKD subtype [23]. Given the important role of hemodialysis in treated ESRD patients, maintaining vascular access via arterio-venous fistula (AVF) is critical. Interestingly, Chen et al. identify two single nucleotide polymorphisms in the angiotensin II receptor 1 that are associated with AVF malfunction [24]. In diabetic patients on hemodialysis, glycated albumin was shown to be a more accurate measure of glycemic control than HbA1c [25].

Emerging therapeutic strategies to attenuate CKD are also addressed. Anemia is a profound complication associated with CKD and two papers in this Issue address therapies for this comorbidity.

CKD-associated anemia is treated with recombinant human erythropoietin (rHuEPO); however resistance often develops limiting therapeutic effectiveness. In the rat 5/6 nephrectomy of CKD, resistance was shown to correlate with renal hypoxia, inflammation and fibrosis [26]; this could stimulate research into adjuvant therapies to treat anemia. In clinical studies, darbepoetin α (DA) and continuous erythropoietin receptor activator (CERA) have similar effects on hemoglobin levels in pre-dialysis CKD patients [27]. Imig and coworkers present compelling data that an omega-3 fatty acid metabolite—19,20-epoxydocosapentaenoic acid—prevents fibrosis in the mouse unilateral ureteral obstruction model, presumably by reducing epithelial-to-mesenchymal transition (EMT) [28]. Using a combination of in vitro and in vivo approaches, it is shown that metformin may be protective against renal fibrosis via inhibition of ERK signaling [29]. The use of angiotensin-converting enzyme inhibitors (ACEI) with atorvastatin may be renoprotective in male patients with coronary artery disease, assessed by GFR [30]. Finally, the role of mTOR inhibitors as therapeutic agents for the treatment of non-clear cell renal cell carcinoma, diabetic nephropathy and renal transplantation, with emphasis on the mechanistic findings underlying the renoprotective effects, is reviewed [31].

The 15 publications in this Special Issue summarize the significant amount of progress that has been made in our understanding of issues surrounding CKD. Importantly, these papers also provide direction for future studies to combat the disease. I wish to thank all the authors for their contributions and the staff at the *International Journal of Molecular Sciences* for their work on this Special Issue.
